# On Dipsomania

**Published:** 1858-04-01

**Authors:** David Skae

**Affiliations:** Physician to the Royal Edinburgh Asylum for the Insane


### Art. VII.—ON DIPSOMANIA.

BY DAVID SKAE, M.D., F.R.C.S.,

*Physician to the Royal Edinburgh Asylum for the Insane.**

Read at the Medico-Chirurgical Society, January 20th, 1858.

When Dr. Peddie read his very interesting and valuable paper on this subject to the Society, at its last meeting, I intended to have made some observations; but the lateness of the hour when the paper was concluded and my seniors in the profession had spoken, and the fear of hazarding rashly, in an unpremeditated and hurried speech, opinions upon a subject of such difficulty and delicacy, determined me to forbear, and to offer these opinions to the Society in the form of a written communication. This communication I beg you will consider not as a reply to or a criticism on that of Dr. Peddie, but as a sequel to his paper,—as a contribution to the solution of the difficulties of dealing with this disease, such as may occur to one occupying *my point of view*, that of the superintendent of a large asylum, where I have had daily practical experience with all the varieties of the disease, and all the difficulties of treating it, for a period of nearly twelve years.

It appears to me that very much of the difficulty of dealing with the question of the isolation of the insane drinker from society disappears, when we have a clear and precise idea of the distinction between the ordinary drunkard and the insane drunkard—between the habit and the disease. To have this, and to estimate properly the mode of treatment and the legality of restraint or isolation, we must be familiar with the differences we meet with in the habits of sane drunkards, and the various features which distinguish the numerous groups or varieties of those who drink because they cannot help it,—from an incontrollable and morbid appetite.

At the risk of repeating some of the valuable descriptions given by Dr. Peddie, I must briefly point out a few of the distinctions referred to, in order to clear the way for the remarks which I have to offer on the treatment of this disease.

A man is a drunkard who gets drunk at the festive board—who seeks occasions for getting conveniently drunk without interfering with his ordinary occupations—who takes a few days' drinking at a time, when he has plenty of money, and returns to his duties and employment when he has finished his superfluous cash and his "ramble," or "spree," as it is called among the

* Dr. Skae has kindly permitted us to transfer to our pages his valuable paper from the March number of the "Edinburgh Medical Journal."

humbler classes in Scotland. Some men, on the other hand, get systematically and regularly drunk every day after dinner, some every night before going to bed, and will perform their daily duties with propriety and efficiency. There is a large class who drink continuously, and consume large quantities of stimulants, and seem to have a constant craving for them, which they satisfy by constant potations; yet they do not come under the denomination of insane drinkers; they exercise a certain amount of self-control, a sufficient amount of restraint upon their appetites not to allow the quantity which they drink to affect their aptitude for business, or driving a good bargain. These are the decorous drunkards of society, and some of them are perhaps the hardest drinkers of any.

The effects of intoxicating drinks vary very much, as is well known, in different individuals, according to the temperament or some peculiarity in the constitution. Some men get hilarious, and others stupid; some become amorous, and others maudlin in their cups; and some, again, become pugnacious, and even dangerous. It has been often observed, that men who have suffered from severe blows or injuries of the head become very violent, and even maniacal, when intoxicated. Such persons as these frequently come under the notice of the legal authorities, for acts of violence committed while intoxicated. But in these cases, if the individual has voluntarily become intoxicated, knowing that in that state he is liable to be dangerous, he is justly held amenable to punishment for acts of violence committed when drunk. The responsibility of a person of this kind for any of the consequences of his getting drunk, knowing what *may* ensue, has been compared to that of a person firing a gun out of a window into a crowded street, who would unquestionably be punished for the effects which resulted from his folly.

It would be a tedious task to describe all the different effects and kinds of drunkenness. It may suffice to say, that in all cases of mere drunkenness, the characteristic feature, as distinguishing them from insane drinking, is the preservation of the power of *self-control.* In the mere drunkard, there is a method in his madness; it is a systematized or regulated, or at least a voluntary habit. A morbid craving may be, and is generated, after drunkenness becomes a habit; but it is regulated, as to the time, and mode, and extent, and frequency of gratifying it, by certain considerations,—such as a regard to outward decency, to the duties of life, or to the calls of business. And it continues to be so regulated, perhaps by a very low standard, and by one which is gradually made lower and lower; but still the habit is regulated and controlled as long as the person is *merely a drunkard,* and until the habit degenerates into a disease.

The principal, the diagnostic feature of this *disease*—that of insane drinking, dipsomania, or oinomania—is, on the other hand, the absence or loss of the power of self-control or self-regulation. Persons affected with this form of *moral insanity* do not drink from the pleasure which the social board affords, but, on the contrary, will not unfrequently preserve a certain amount of decorum while in company. Neither do they drink for the pleasure which the wine gives them; they will drink any kind of intoxicating stuff. Nor do they drink at a convenient time and place, and only occasionally; they drink as often as they can, and whenever they can, and as much as they can; their craving for stimulants is incessant and incontrollable; no considerations of self-respect, no regard to public opinion, or common decency, or domestic ties, or religion, or the certainty of impending ruin, degradation, or even the fear of death, can prevent their drinking till they can drink no longer. Such persons will often deplore their fatality, their inability to control their desires, and will say, with tears in their eyes, as some have done, that if hell were yawning at one side of them, and a bottle of brandy standing at the other, they would drink, although the next moment they were to be thrown into the bottomless abyss. In fine, such persons drink because *they cannot help it;* and if they really *cannot* help it, they must be regarded as no longer responsible agents—as therefore insane, and proper objects for being restrained and protected against themselves.

The *loss of self-control* is, indeed, the most essential condition of almost all cases of insanity. The most constant symptoms of insanity are those referable to altered affections, perverted desires, and morbid propensities. *Delusions,* so much dwelt upon by some writers, particularly among legal authorities, as essential to insanity, are but its accidental concomitants, like hallucinations of the senses. Every form of madness, from acute mania down to dementia, may be met with where there are no delusions. In mania, there may be a morbid exaltation of all the emotions and passions, over which self-control is entirely lost, and the individual riots in unrestrained laughter, singing, anger, destructiveness, and violence. The loss of control may extend to the thoughts and ideas, which succeed each other with extraordinary rapidity, and give rise to incessant talking and incoherence. In partial insanity, one particular emotion or desire may be morbidly excited, and we may find a deep melancholy and depression without any delusion—a hopeless but abstract gloom, leading, by the total overthrow of the power of self-control, to an act of suicide. There may be, as has been fully established by numerous well-authenticated cases, a morbid impulse to destroy life, without any delusion; an impulse over which the affected person loses all control, and under which he commits some homicidal act. The morbid excitement of the sexual appetite only becomes insanity when the person loses self-control, and the victim of satyriasis or nymphomania outrages all considerations not alone of morality, but of ordinary decency and the common instincts of humanity.

It is, in fact, the loss of control over morbidly excited passions, whether of an exalted or depressing kind, that constitutes *insanity*, and distinguishes it from ordinary excitement or depression. So it is with insane drinking; it is the loss of control over the morbid appetite that distinguishes it from ordinary drunkenness, and constitutes it a form of *moral insanity*.

I prefer the term moral insanity, in describing this disease, to the terms *oinomania* or *dipsomania*, for three reasons: *First*, because neither of these names conveys a correct idea of the disease, which is not an insane thirst, nor a thirst for *wine* only, but may consist in a morbid craving for stimulants or narcotics of any kind; *Secondly*, because it is under the denomination of moral insanity that this disease is recognised by the Commissioners in Lunacy for England; and, *Thirdly*, for the most important reason of any—namely, that the craving for stimulants is generally only *one* of many symptoms of moral perversion which characterize the disease, and which it is of great importance to recognise, as they render the duties of the medical attendant comparatively easy, when they are met with in any particular case.

Of these other symptoms of moral perversion, the most common is the habit of lying. In almost all cases of this variety of moral insanity, there is a total disregard for truth. Such persons are singularly mendacious. They will resort to every possible device to procure stimulants, to excuse their conduct, to deceive their friends and medical attendant, and will display an ingenuity and fertility in deceit which is truly marvellous. They will become faint, or be in agony with toothache, or tic, or cramp in the stomach, or colic, or dysmenorrhœa, or they will take diarrhœa or hæmorrhage, and be on the verge of dissolution, unless brandy, wine, or opium is administered. They will pawn every available article of dress, or furniture, or jewellery. They will borrow from servants or cabmen. They will get whisky smuggled home with their clothes from the tailor or the laundress. They will evade the most vigilant surveillance, and tell the most deliberate falsehoods in their attempts to deceive, solemnly appealing to God for their truth. When shut off from the ordinary sources of stimulation, they will sometimes resort to almost anything in order to relieve their craving. I have known a young and delicate lady, after being prevented getting wine or spirits, and deprived of red lavender, lavender water, and eau de Cologne, take creosote, vinegar, vitriol, and tobacco.

Allied to this disregard of truth is the total denial, often insisted upon by such persons, of their habit of over-indulgence. They will very frequently disavow most solemnly having ever exceeded the bounds of strict temperance in their use of stimulants ; and, if admitting at all that they ever have been the worse for drink, they will blame some other person or circumstance as the cause of it. The wife will blame the husband ; the husband, the accident of meeting on one particular occasion some one, or being engaged in some circumstances which rendered it unavoidable. And in this way, one or two undeniable overt acts of intemperance are conceded, when it cannot be avoided, while the habit or the loss of self-control is generally indignantly denied, except at moments of remorse.

Accompanying this disregard of truth, there are often other indications of moral perversion in the insane drunkard, such as extreme licentiousness, or a propensity to theft, or a delight in fomenting quarrels and creating mischief by leading other parties into trouble.

Again, this disease is very frequently met with in persons presenting certain mental peculiarities which are natural to them —that is, congenital. It is by no means uncommon to find it develop itself in youth who have always been regarded as having a want about them, to use a vernacular phrase. They have never made progress in their education like others of their age—have never, perhaps, been able to learn to spell, and so forth ; and, although illiterate and ignorant when they come to manhood, are often very vain, and at the same time credulous and silly. Or, again, they have been characterized, even from childhood, by certain perversities of disposition, such as a delight in cruelty to animals, disregard of personal appearance, love of solitary and gratuitous destructiveness, extreme obstinacy and selfishness, or a violent and mutinous temper upon slight occasions.

Lastly, the disease is very frequently hereditary ; and it will be found, on inquiry, that a grandfather, or father, or mother, or one or more brothers, have died of delirium tremens, or have otherwise shown the fatal propensity. The constancy with which the hereditary predisposition to this disease is transmitted is most remarkable, and, in a vast majority of cases, can be traced either through the maternal or paternal side of the family.

To illustrate the truth of these remarks, I have analysed the record of 86 of the cases of this form of moral insanity which I have had under my care, of which 20 were females and 66 males. I find, as the result of these comparisons, that of the 20 females one-half presented natural peculiarities in their mental constitution, being either of weak minds, of very violent and incontrollable passions, or subject to hysterical attacks of great violence, combined in some instances with great moral depravity.

Of the males, 30, or nearly one-half, were naturally of weak mind, or presented some mental peculiarity, such as silly vanity, general depravity of disposition, and, in some cases, considerable talent, but combined with eccentricity of some marked peculiarity. In both sexes, the disregard of truth was a common feature in most cases.

In regard to hereditary predisposition, it is difficult generally to trace this in a public hospital, partly because many cases come in without any information regarding them at all, and partly because the friends, when they have friends, are generally very solicitous to deny or conceal the existence of a hereditary taint in the family, and will not unfrequently positively deny that any other member of the family is insane, while, perhaps, at the time there may be a mother in one asylum and a sister in another. Notwithstanding these difficulties, I find the hereditary predisposition distinctly traced in 32 cases out of the 86.

Of the females, this hereditary taint was found to exist in 10 out of 20. One had a brother insane; one a mother half-witted; one had two cousins who died of intemperance; another had a father and mother who were both intemperate; another had a father, and another a father and brother, who were hard drinkers; three had mothers who died from the effects of intemperance; one had two sisters, both prostitutes and drunkards, one of whom committed suicide; and another had a brother, father, and mother all intemperate, and the latter of whom committed suicide.

Of the males, two had brothers affected with the same disease in the asylum, and three brothers who had the same disease, but were not sent to any asylum; one had repeatedly attempted suicide, and inherited, with his whole family, a loathing of life, which at times affected all the members of his family. Four had brothers who suffered from other forms of insanity; two had each two brothers insane; three had insane sisters; two had drunken fathers; one a drunken father and brother, and one a father and grandmother drunkards; and of several others it was admitted that insanity was in the family, although the relationship of the members affected was not ascertained.

Before discussing the question of treatment, and isolation in an asylum or special establishment, it is necessary to complete this sketch by enumerating briefly the varieties of this form of moral insanity, and the causes which induce the disease.

Its varieties group themselves naturally into three divisions,—the *acute*, the *periodic*, and the *chronic* forms of the malady.

Under the head of *acute*, I would include all those cases of incontrollable drinking which occur to persons of previously temperate or regular habits, but in whom this insane craving has been generated under the influence of some accidental cause,—

such as the novelty and excitement of a new sphere of duty, and the temptations of new associates and habits, all combining to lead to intemperance; or the depressing effects of some overwhelming calamity, or of some debilitating accident, or disease, or other agency, such as excessive hæmorrhage, protracted nursing, fever, and such like. It is well known that the use of stimulants, begun and indulged in under any of these circumstances, has gradually merged, in many instances, into an inordinate and incontrollable craving, and an insatiable and destructive use of them.

Cases of this kind are, however, if taken in time and treated judiciously and firmly, generally curable without recourse to any step for depriving the patient of his personal freedom.

*Periodic* or *recurrent* attacks of the disease are generally dependent upon some constitutional or hereditary peculiarity. Sometimes they occur at the critical age in females, and preserve a periodic form coinciding with the menstrual period. Sometimes they arise from injuries of the head. In many cases the cause is obscure.

Cases of this kind are less amenable to treatment than the former, particularly where there is a hereditary predisposition. They hardly justify confinement, however, or isolation from society. Where this has been tried, it has generally been found that the incontrollable impulse comes back at its accustomed period, when the imposed restraint has been removed. Nor does this form of the disease interfere materially, in many cases, with the duties of life. Many individuals have distinguished themselves in literature, or in professional or mercantile life, who have been known for a long term of years to retire periodically into the privacy of their own chamber, and, after indulging this morbid appetite to satiety for a week or two in their voluntary seclusion, to reappear again on the stage of life, and pursue their usual avocations with credit and success. Such cases are perhaps rare, and more frequently it happens that the recurrent or periodic form gradually degenerates into the chronic variety of the disease, the intervals becoming shorter and the attacks longer, until the intervals cease altogether, and a chronic disease or a fatal issue ensues.

It is the *chronic* form of this disease which is the least curable and most troublesome to manage. Here the craving for stimulants, brought on perhaps by indulgence and irregular habits, operating upon a constitution hereditarily predisposed, becomes constant, insatiable, and incontrollable; and the daily or hourly indulgence suffers only now and then a temporary check by illness induced by it, by attacks of delirium tremens, or outbursts of mania.

I believe it is agreed by all parties, that this form of the disease can only be treated effectually by prolonged and complete abstinence from all stimulants. This has been attempted in two ways: First, by boarding the patient in some house where, in consequence of its remoteness from places where ardent spirits or other stimulants can be procured, or where, by the stringent rules of the house, and the watchfulness of those connected with it, a barrier may be placed against attempts to gratify the morbid craving. Of the first kind is the boarding-house mentioned by Dr. Christison, in Skye, where for many years patients of this kind have been sent. Of the latter is the House of Refuge in Edinburgh, which has been used for a number of years as a reformatory school for patients of the humbler classes affected with this disease. In addition to these houses, a number of individuals—clergymen, medical men, and others, in different parts of the country—have been in the habit of taking boarders affected with this malady, with the view of curing them. In none of these places can the principle of treatment be efficiently carried out. The parties having charge of the patients have no legal authority to detain them, or to interfere with their personal freedom; and the consequence is, that they either leave before a sufficient length of time has elapsed to do any good, or they evade the surveillance under which they are placed, and manage to keep up the craving for stimulants by a constant system of smuggling and secret indulgence. It consists with my experience that this has been the result in all the institutions and houses of the kind mentioned, which I know of, in this country; and I believe every one will agree with me, who has had much experience in the treatment of this deplorable malady, that, in its chronic and confirmed form, complete abstinence from stimulants can only be effected by depriving the patient of his personal freedom.

In the present state of the law, this can only be done by obtaining a warrant for the detention of the patient in an asylum for the insane. And this method of treatment is now very commonly adopted in well-marked and obstinate cases. I think some medical men, particularly in the country and in provincial towns, still entertain doubts as to the propriety of granting certificates of insanity in such cases. But the scruples of such are gradually wearing away, as the disease becomes better known and more fully recognised as a variety of moral insanity. It is now found, accordingly, that in all the asylums of England, Ireland, and Scotland, such cases are constantly admitted, and regarded both by the medical superintendents, the Commissioners in Lunacy, and the sheriffs of our counties, as the proper objects of asylum restraint.

Lest there should be any doubt in the minds of any one as to the legality of certifying such patients to be insane (and I think some doubts were expressed by Dr. Peddie, and by others, in the discussion which followed his paper), I may mention that in one or two cases sent to the asylum under my care, the medical certificates distinctly set forth that the patients laboured under an incontrollable or insane craving for stimulants, and the sheriff granted warrants upon these certificates. In one case sent to the asylum by Dr. Christison and Dr. Douglas Maclagan, in 1854, the medical certificate was as follows:—"We consider his case to be a very aggravated one of insane propensity to drinking; that he appears for a long time past to have been never sober, except under compulsion; and therefore we consider him to be insane, and a fit subject for confinement in a lunatic asylum, both for the safety of himself and others, and likewise for treatment."

Under the new Lunacy Act for Scotland, the medical certificates must now contain those facts observed by the medical men themselves, and those communicated to them by others, on which their opinion has been founded as to the insanity; yet no difficulty has as yet been experienced in obtaining warrants for the confinement of such patients in asylums. In England these forms of certificates have been in use for years, and I have never understood there was any difficulty in sending such cases to asylums there.

In the First Report of the English Commissioners in Lunacy, issued in 1844, the form of insanity under discussion is distinctly recognised and very graphically described.

The term "moral insanity," they say, "is used to designate a form of mental disease in which the affections, sentiments, habits, and, generally speaking, the moral feelings of the mind, rather than the intellectual faculties, are in an unsound and disordered state. The common distinctive character of all these cases is of a negative kind—viz., that the faculties of the understanding remain apparently unimpaired, and that no delusive impression can be detected in the mind of the patient, which may account for the perversion of his moral disposition, affections, and inclinations. Cases of this kind were formerly looked upon as unaccountable phenomena. They are, however, now recognised as a distinct form of mental disorder in nearly all the public asylums. They are characterized by a total want of self-control, with an inordinate propensity to excesses of various kinds—among others, habitual intoxication. This is often followed by an attack of mania, which, however, speedily subsides when the patient is confined, but is generally reproduced by the same exciting cause soon after he is discharged."—(*Report*, p. 108.)

In the Eighth Report of the Inspectors-General for Ireland, 1857, the disease is specially, and with even more distinctness, recognised and defined.

"As formerly," they say, "so within the last year, cases of *moral madness*, originating in drink and dissipation, have been frequently admitted into private licensed houses. Some of them, discharged after a few months' confinement, were not since readmitted, whilst others have been brought back. These latter cases are most perplexing: even after the lapse of a few days, the salutary effects of control are visible in their regard; once free, however, they become the mere children of impulse, reckless of personal respect, regardless of the value of money, and scorning even decency itself. Rational in conversation, and most plausible in manner, within the asylum, their conduct is displayed out of doors in a series of the most irrational actions. It is painful to keep such parties confined, but still more so to let them run at large to certain destruction. As an illustration, we may adduce the case of a lady, at present in confinement in a private licensed house, who has been admitted and discharged four or five times within our knowledge, and who, when at liberty and mistress of her allowance, spends it in one continued orgie of drink and dissipation. We may here observe that a characteristic of this class is, at all times, *an utter disregard of truth,* together with an unceasing desire to impose upon the credulity of their hearers by the most specious pretensions to sense and wisdom, and the most solemn promises that a future morality would efface the errors of their past life."—(p. 24-5.)

With these statements before us, it cannot be doubted, on the authority of the highest judges in the country, that persons affected with the moral insanity I have described are proper objects for confinement in asylums. A consideration of the general moral perversion referred to by the Irish inspectors, of the mental imbecility apparent in so many cases, and of the hereditary tendency so commonly to be detected, will tend to confirm this view of the subject in the minds of any who may still hesitate to adopt it.

Dr. Peddie referred to the legal definitions of insanity, as laid down by Hume and other distinguished legal authorities, in order to show that this form of the disease did not come under the legal definition of insanity, and that medical men, by inference, might not be legally justified in certifying a dipsomaniac to be insane. The same argument would apply to all other forms of *moral insanity;* and if a medical man were liable to prosecution or penalties for certifying a dipsomaniac to be insane, he would be equally so for granting certificates of insanity in a case of homicidal or suicidal madness, or in one of nymphomania, or indeed in any case of partial insanity where there was no delusion.

But I conceive the legal definitions of insanity, as laid down in legal works, have nothing whatever to do with the distinctions recognised by physicians as rendering a person insane with a view to his treatment in an asylum. The legal definitions have a legal bearing only, and refer to the kinds of insanity recognised in our courts of law as exculpating from crime, or as nullifying wills or other civil acts. The certificates of insanity granted for the purpose of confining a patient in an asylum, and the warrant of the sheriff, do not make him legally insane in these respects; they neither disqualify him for civil acts, nor exempt him from criminal responsibility. A will executed by an inmate of an asylum might be held to be good, on proof of his not being affected with that kind or degree of insanity which incapacitated him in the eye of the law from disposing of his property; and a person committing a criminal act, an act of homicide, might not claim exculpation in a court of law on the mere plea of being in an asylum, unless the insanity were such as to prevent him from distinguishing right from wrong in the act which he committed.

In the same way, the appointment by the Court of Session of a *curator bonis* to a party, on the ground that he was insane and incapable of managing his own affairs, would not of itself be sufficient to make that person a proper object for confinement in an asylum, nor would it exculpate him from a charge of assault, or any other criminal act.

Lest these opinions may be cavilled at as loose or conjectural, I think it right to add a few remarks in explanation, with one or two references to facts in support of them. I by no means assert that confinement in an asylum does not affect a person *at all* in relation to his civil rights or responsibilities. It affects him to this extent, that it affords an *à priori* presumption that he is insane, and therefore incapable—*e.g.*, of executing a valid will, or irresponsible for a crime. But that is only a presumption, which can be set aside by evidence. The burden of proving insanity, in such a case, is shifted to the opposite party. When a man, not in an asylum, executes a will or commits a crime, the presumption is that he is sane till the contrary is proved; and the parties wishing to set aside the will, or to exculpate from crime, must prove insanity. But, on the other hand, if a will were executed, or a crime committed, by a person in an asylum, the presumption would be that the person was insane, and that the will was invalid, or the person irresponsible, until the contrary was proved. And in this case the parties wishing to establish the validity of the will, or the responsibility for crime, would require to establish by proof that the person enjoyed a lucid interval at the time, or that he was sufficiently sane to be able to dispose of his property, or to distinguish right from wrong in the criminal act which he committed. That these are not mere theories could be established by numerous references. Many disputed wills have been declared to be valid, although made by persons who were undoubtedly and confessedly insane, to a certain extent, at the time at which they were executed; and many have been convicted of criminal offences, and punished for them, who were insane at the time of their commission, but whose insanity was not, in the opinion of judge and jury, of such a nature or degree as to render them irresponsible for their acts. I shall cite only one or two cases in corroboration of these statements. In the case of Cartwright *v.* Cartwright and Others, the testatrix wrote her will with her own hands, *loosened from their ligatures for the purpose;* and, while writing it, she was observed frequently to leave off writing, throw torn pieces of paper into the fire, and walk about the room in a wild and disordered manner. Yet the will was held to be valid, because it bore no marks of agitation or insanity, and was consistent with her attachments and impressions when sane.* In another case (that of Coglan), the person made his will *while in an asylum;* but, on proof that at the time he made it he was as competent to converse on the subject of testamentary dispositions as before, and that he had before his insanity intimated his intention of leaving his property as he did in the will, it was held to be good.†

In regard to the responsibility for crimes, need I refer to the well-known case of Bellingham, who was convicted and executed for shooting the Hon. Spencer Perceval; to that of Howison, executed in Edinburgh in 1832, for murdering the woman Geddes; or to many others, in proof that partial insanity does not exculpate from the guilt of murder in every case? And surely it will not be questioned that Bellingham and Howison might have been justly detained in asylums, and indeed ought to have been in asylums, in order to prevent the acts which their insanity prompted them to commit. In the case of Thomas Bowler, an epileptic maniac, tried and executed for shooting at William Burrowes,‡ a Commission of Lunacy finding the prisoner insane for some months previous to the commission of the act was actually produced in his defence, yet he was convicted. These facts, and many others of a similar kind which might be cited, leave no doubt, I think, that the legal definitions of insanity do not affect the question of committal or detention in an asylum for the purposes of treatment, but refer specially and only to the capacity of the person for civil acts, or to his responsibility for acts of a criminal nature.

I conceive that a man is insane, and legally so, for the purposes of confinement in an asylum, if two medical men certify on soul and conscience, and do not wilfully or falsely so certify, that he is insane, and a fit object for an asylum, and if the sheriff grants a warrant accordingly, whatever the definitions of insanity given by Blackstone, Erskine, Hume, or the twelve judges may be.

I may add, that petitions for liberation, on the ground of

* Shelford "On the Laws concerning Lunatics," &c., p. 382.

† Ibid. p. 387. ‡ Ibid. p. 590.

wrongous detention, have been uniformly, and in innumerable instances, forwarded by me, from parties labouring under this disease, to the sheriffs, to the Lord Advocate, and the Secretary of State; and in no instance have any of those authorities ordered the release of such patients, on the ground that they were improperly or illegally detained in the asylum. In one case, referred to by Dr. Peddie, that of a gentleman confined under the 4 and 5 Vic., c. 60, as a dangerous lunatic, whose case was confessedly one of moral insanity of the kind before us—one of insane drinking, there was an appeal made in due form to the High Court of Justiciary for liberation, yet the Court refused to interpose or reverse the order of the sheriff.*

It cannot, therefore, be doubted, on a review of such facts, that persons affected with this form of moral insanity may be legally and properly consigned to asylums, and detained there for treatment.

The principal ground, however, upon which it is contended that some new legislative enactment is required for the treatment of the cases of moral insanity under consideration is, that they *cannot be detained long enough in asylums*, under the present laws, for the purpose of effecting a cure of the disease. In regard to this point, I must confess that the difficulties I have had chiefly to contend with are such as could be obviated by no new act of legislation. Such patients, when removed too soon, have been so removed almost invariably by their own relatives, or on the advice of their own medical attendants. When they recover from the immediate effects of their habits of uncontrolled drinking, they appear so sane, they make so many plausible promises, and they are so full of self-reliance, that they generally succeed in persuading their friends that they ought to have a trial; or they try the art of intimidation—threaten to quarrel with their friends and cut them off without a shilling in their wills, unless they take them out, or threaten the medical attendants who consigned them to the asylum with actions of damages, unless they are immediately liberated, and so succeed in getting the one

* Since the preceding pages were written, an important case, bearing upon the subject, has been decided in the English courts, before Lord Campbell and a special jury. In this case (the Queen *v.* Armstrong), the lady had been found a lunatic under a commission; and the issue tried was whether she was justly found to be of unsound mind, and incapable of managing herself and her property. The evidence was to the effect that, although not of strong mind, she had no delusions, but had a craving for stimulating drinks which she could not control, and she said she would have wine if she died for it. She would have it for all her father, the doctor, her husband, or God himself. When she was at large, she acted honourably in her monetary dealings, but drank to a great excess. Lord Campbell agreed in the law that intemperate or immoral habits alone would not be enough to constitute insanity; but, after reviewing the whole evidence, the jury found that she had been of unsound mind at the time of the inquisition, and was now "incompetent to manage her own affairs on account of her habitual drunkenness."—*Times*, 11th and 13th February.

party or the other to recommend their liberation, rather than encounter the threatened alternatives. I cannot recall to my recollection a single instance in which any such case of moral insanity has been ordered out of confinement by the legal authorities contrary to my advice, and before I considered the patient was justly entitled to a trial. I am quite willing to admit that, in many of the cases I have had under my care, the sheriff might have intimated his opinion that they ought to have a trial by being set at large, had his interference not been forestalled by that of his friends; but I feel confident that if such cases, by premature liberation, had relapsed and been sent back, their detention would not have been interfered with during a very considerable period of probation. And admitting, as I do, the necessity of a pretty long period of confinement, extending even to one or two years, or even more, for the cure of confirmed and chronic cases of this moral insanity, I cannot but admit at the same time the justice and propriety of giving patients of this class a trial, after their first confinement, at the end of three or six months, if they give anything like a reasonable hope of doing well, and if their powers of self-control have been tested with favourable results. It has been my practice, and I believe it has been that of the physicians of other chartered asylums, to test the powers of self-control of such patients, after they have been sufficiently weaned by abstinence from their morbid cravings — to permit them to go beyond the asylum on their parole, to visit their friends, to spend a day or two with their relatives, or even to lodge for a week or two in the neighbourhood; and if they display, under those circumstances, the ability to control their appetites and regulate their conduct, then I consider myself bound to liberate them; while, on the other hand, if they break through the restraints imposed upon them by their own voluntary pledges, and are unable to keep their promises and purposes of temperance, I consider myself entitled to prolong their period of confinement, and, in doing so, I have never yet been interfered with by the arm of the law.

The practice in other parts of the empire may be different— the views which some sheriffs, or the views which our newly-appointed Commissioners in Lunacy may take of such cases, may be different; but I do think, as the real nature of this deplorable disease becomes more fully known, the administration of the existing laws must expand, so as to permit all that can be properly and wisely asked as to the duration of time required for the effectual treatment of such cases, with a view to their complete cure of a morbid, and incontrollable, and self-destroying appetite.

There is, however, another objection to the management of such cases in asylums,—and that is, the natural repugnance felt by the friends of patients, and by the patients themselves, to their being associated with persons affected with other forms of insanity. It is considered very hard that persons who, in a few days after their isolation, recover apparently the use of their reason and all their other faculties, should be compelled to associate with raving maniacs, with the fatuous and demented, and with melancholy or violent companions.

To meet this difficulty, and the more serious one already discussed—viz., that such persons cannot generally be detained long enough in asylums to effect a radical cure of their disease, Dr. Peddie proposes that an effort should be made to obtain some legislative enactment for the management of such cases, and that separate establishments should be provided for their treatment, under the powers to be granted by such an enactment. I am far from saying that this is not desirable; on the contrary, I believe it would be a great boon to society, and would remove most of the difficulties which must continue to be experienced, under the present law, in the treatment of moral insanity.

I fear, however, the prospect of any legislative Act on the subject is very distant and very problematical. The difficulty of dealing with it, the fear always dreaded of interfering with the personal liberty of the subject, the apprehension that the operation of any such Act might extend to mere drunkards, or might be otherwise improperly used, must operate, I fear, at least for many years, to prevent any attempts to legislate regarding this disease.

My object in this paper, therefore, has been to show that, under existing laws, there are facilities for the isolation and treatment of undoubted cases of dipsomania, which may serve all practical purposes. It is impossible to foretell what the laws regarding the detention of such cases in asylums for a proper term of probation and treatment may be under the administration of the recently-appointed Commissioners in Lunacy for Scotland; but, judging from the tenor of the Report of the late Commission of Inquiry, and from the known character and ability of the medical Commissioners, it is not to be anticipated that a narrow-minded or restricted view will be taken of the nature of such cases, or the necessity of prolonged confinement for their cure.

The English Commissioners, in their first Report, say—"It has been our practice, in cases of this sort, to liberate the patients after a short confinement, if it be the first attack of insanity from this cause, and if he appear to be aware of his misconduct, and to have a desire to reform his habits. In the event, however, of his being confined a second time owing to the same cause, we have felt that his probation ought to continue for a much longer period; and indeed we have felt that great responsibility has rested upon us in such a case, and have at all times very reluctantly—and only after vainly endeavouring to induce the patient's friends to take charge of him—resorted to our power of liberation."—(p. 176.)

I have uniformly found the authorities in Scotland influenced by the same principles; and I have no doubt, as the true nature of this disease becomes more fully recognised, that these principles will continue to be acted upon, and made to reach the requirements of the proper medical treatment of the disease by sanctioning a sufficiently long isolation.

In regard to the second objection to asylum treatment, that can be met at any time, if these observations are correct, by establishing either a private or a public asylum specially devoted to the reception of such cases. The patients would be sent there, of course, under the same certificates and warrants as other cases of insanity; but the institution preserving its distinctive character as an hospital for such cases only, and having, if it were thought proper, a special designation, would not be liable to the objections urged against an asylum for the insane.

In fine, if the disease which we call moral insanity, or dipsomania, is not really a form of insanity, it would be very dangerous to legislate on the subject; but if it is a form of insanity—and of this, I think, *we* have no doubt—the existing laws regarding the insane ought to meet the requirements for its treatment; and, by a liberal and enlightened administration of those laws, I do not doubt they will be found to do so, without the necessity for new legislation. If anything more is wanted, let some enterprising individual, or company, with a moderate capital, license a country-house surrounded with an agreeable landscape, and with grounds affording extended walks or drives, and the amusements of fishing and shooting; let the establishment command all the sources of recreation which a well-arranged asylum possesses; and let it be designated by some appropriate and agreeable name—not "*Asylum*," nor "*Inebriate Asylum*," like the American institution of the kind, which, I believe, did not succeed; let the patients of this class be sent to that sanatorium under a warrant of the sheriff as insane; and let them be treated according to the most approved principles of our art, under the direction of an enlightened physician, with an efficient staff of trustworthy attendants; and let patients be taken at different rates of board, suitable to their means,—and I doubt not most of the difficulties at present felt in dealing with this disease, if not all, will be successfully met, and that such an establishment would soon be distinguished for its usefulness, and prove a valuable source of revenue to its proprietors.

